# Volumetric MR-guided high-intensity focused ultrasound ablation to treat uterine fibroids through the abdominal scars

**DOI:** 10.1186/2050-5736-3-S1-P88

**Published:** 2015-06-30

**Authors:** Zhu Ying, Bilgin Keserci, Xuedong Yang, Xiaoying Wang

**Affiliations:** 1 Peking University First Hospital, Beijing, China; 2 Philips Healthcare, Seoul, Republic of Korea

## Background/introduction

Magnetic resonance (MR)-guided high intensity focused ultrasound (HIFU) is an emerging therapy technique using focused ultrasound to heat and coagulate tissue deep within the body, without damaging intervening tissue. However, massive abdominal scar tissues were considered as relative contraindications as the higher energy absorption of the scar tissue might result in skin heating at the site of the scar.[1] Obstruction in the near-field of the focused ultrasound beam, such as indeed extensive abdominal wall scar tissue could lead to increased absorption of acoustic energy and skin burns.[2] In some conditions, it is possible avoid the ultrasound beam passing through the scar tissue by tilting the transducer or filling the bladder.[3] Yoon *et al*. started to use the scar patch to blocking the beam, which would reduce the risk of skin burn and enlarge indications with point by point technique.[4] In our preliminary study, we used the scar patch in three patients, one with transverse incision, and the other two with longitudinal incision using volumetric technique.[5]

## Methods

MR-HIFU treatment was performed by stepping through several treatment cell ablations with cooling times between each sonication using a Philips Healthcare clinical HIFU platform integrated into a 3 T Philips Achieva MR scanner. The scar patch used in this study was made of isolation polyethylene foam (1.5-mm-thick Cell-Aire; Sealed Air, Elmwood Park, New Jersey) covered with a double-coated medical tape (9889; 3M, St. Paul, Minnesota). FFE was performed for the scar and scar patch with the following imaging parameters: coronal, TR/TE 3.4/1.74ms, FOV 200mm, slices 29 thickness 1.0mm, Voxel size 1.5×1.5×1.0mm3, TA 1min 18sec. 3D T2WI was performed for treatment planning: sagittal, FOV 241mm, TR/TE 1550/150ms, thickness 1.6mm, slice 150. Voxel size 1.0×1.0×1.6mm3, TA 5min56sec. The MR sequence used for temperature mapping is an RF-spoiled segmented Echo Planar Imaging sequence (EPI-factor = 11, repetition time TR = 37ms, echo time TE = 19.5ms, 121-binomial water selective excitation). Immediately following the treatment, Fat-saturated T1-weighted THRIVE sequence used to for evaluate the volume of the fibroids ((turbo field echo (TFE), 17 axial slices; TR/TE: 500 / 10 ms; slice thickness: 5 mm with 1 mm gap; FOV, 240 X 240 mm; matrix, 320 X 250; flip angle: 90 degree).

## Results and conclusions

In the first case, a 41-year-old woman with transverse incision had a single intramural type 1 fibroid. The volume of the fibroid was 111.98ml. The treatment time was 165min 17sec. Following delivery of multiple sonications to the treatment area, The NPV immediately after treatment was 52.35%. No abnormal areas of enhancement within the subcutaneous tissue or the regions of the scar were identified. In the second case, a 49-year-old woman with a longitudinal incision had multiple type 1 fibroids. The target fibroid was located in the anterior wall. The scar patch was in the middle sagittal plane while the fibroid was located left of the uterus. The volume of the fibroid was 71.17ml. The treatment time was 112min 1sec. The NPV immediately after treatment was 50.32%. Skin heating was mild, and no severe advent event occurred. In the last case, a 43-year-old woman with a longitudinal incision also had multiple type 1 fibroids. The volume of the fibroid was 100.90ml. The fibroid was in the left of the uterus, almost beyond the scar. The treatment time was 100 min 44 sec. The NPV immediately after treatment was 73.21%. Mild skin heating was complained, without severe advent events. The scar patch could effectively avoid heating around the scar tissue (both horizontal and longitudinal), and expand the indication of MR-HIFU treatment of uterine fibroids with volumetric technique.

## 

Written informed consent was obtained from the patient for publication of this abstract and any accompanying images
